# Exercise prevents HFD‐ and OVX‐induced type 2 diabetes risk factors by decreasing fat storage and improving fuel utilization

**DOI:** 10.14814/phy2.13783

**Published:** 2018-07-06

**Authors:** Brittany K. Gorres‐Martens, Tyler J. Field, Emma R. Schmidt, Karen A. Munger

**Affiliations:** ^1^ Exercise and Sport Sciences Department Augustana University Sioux Falls South Dakota; ^2^ Research & Development Sioux Falls VA Health Care System Sioux Falls South Dakota

**Keywords:** Exercise, metabolism, ovariectomy, type 2 diabetes

## Abstract

Previous studies suggest that the loss of estrogens increase one's risk for type 2 diabetes (T2D), and combining the loss of estrogens with a high‐fat diet (HFD) poses an even greater risk for T2D. The extent to which exercise can ameliorate the deleterious effects of estrogen loss combined with a HFD and the molecular mechanisms accounting for the whole body changes is currently unknown. Therefore, we fed female Wistar rats a standard diet or a HFD for 10 weeks. The rats fed the HFD were either ovariectomized (OVX) or their ovaries remained intact. A subset of the HFD/OVX rats also underwent exercise training on a motor‐driven treadmill. Exercise significantly reduced the total body weight gain, periuterine white adipose tissue (WAT) weight, hyperglycemia, and hyperinsulinemia. Additionally, the ability to store fat, as measured by lipoprotein lipase (LPL) in the WAT, was increased in the HFD/OVX group; however, exercise reduced the LPL levels. Furthermore, the combination of the HFD with OVX decreased the WAT citrate synthase protein level, which was increased with exercise. These data suggest that even during the combined HFD/OVX physiological state, exercise can decrease several risk factors associated with T2D, decrease fat storage, and increase fuel utilization.

## Introduction

Type 2 diabetes (T2D) is characterized by insulin resistance and high blood glucose. In healthy individuals, insulin promotes glucose uptake from the blood and into cells (Alessi et al. 1997; Kane et al. 2002; Sano et al. 2003; Gonzalez and McGraw 2006; Peck et al. 2009), most notably, the skeletal muscle cells and white adipose tissue (WAT) (Klip et al. 1990; Bjornholm and Zierath 2005). However, with disruption of the insulin signaling pathway during T2D, blood glucose remains high. This hyperglycemia is a clinical indicator of T2D and further spurs hyperinsulinemia, another clinical indicator of T2D.

Several studies suggest that postmenopausal women have a greater risk for T2D compared to premenopausal women (Lindheim et al. 1994; Lynch et al. 2002; Moreno et al. 2010). Following menopause, insulin insensitivity and glucose intolerance occurs, along with weight gain (Pfeilschifter et al. 2002; Sites et al. 2002; Carr 2003). As weight gain, and specifically abdominal obesity, is the number one risk factor for T2D (Kissebah and Peiris 1989; Chan et al. 1994; Despres 2006), many animal studies induce T2D by feeding rodents a high‐fat diet (HFD) to stimulate weight gain. However, previous studies suggest that female rodents demonstrate protection against HFD‐induced T2D (Corsetti et al. 2000; Coatmellec‐Taglioni et al. 2002; Yakar et al. 2006; Hong et al. 2009), with benefits including no significant weight gain (Coatmellec‐Taglioni et al. 2002), weight gain but to a lesser extent than male rodents (Hong et al. 2009), or weight gain without insulin resistance and glucose intolerance (Yakar et al. 2006). These studies suggest that the presence of estrogens may provide protection against T2D.

To model the postmenopausal state in rodents, removal of the ovaries (ovariectomy; OVX) is often performed. Like postmenopausal women, OVX in rodents results in total body weight gain (Yakar et al. 2006; Nunez et al. 2007, 2008; Hong et al. 2009), total body fat gain (Nunez et al. 2007), and impaired insulin sensitivity and glucose regulation (Kumagai et al. 1993). A HFD combined with OVX further increases weight gain in female rodents compared to OVX alone (Yakar et al. 2006; Nunez et al. 2007, 2008), and the protection from T2D that high‐fat fed female rodents demonstrate over male rodents diminishes when combining the HFD with OVX (Hong et al. 2009) or after the rats become acyclic (Gomez‐Perez et al. 2008). These studies suggest that the loss of estrogens may increase the risk of T2D in females, particularly when combined with a HFD.

Exercise is an established means to prevent and ameliorate obesity and T2D (Ploug et al. 1990; Wake et al. 1991; Lund et al. 1995; Kennedy et al. 1999; Sriwijitkamol et al. 2006; Winder et al. 2006; Goedecke and Micklesfield 2014). However, whether or not, and to what extent, exercise is a viable prophylactic treatment for T2D when combining a HFD with estrogen loss remains unknown. Therefore, this study compares the whole body and cellular changes in female rats fed a HFD in the presence and absence of ovarian estrogens and examines the extent to which exercise can ameliorate the effects of the HFD/OVX‐induced T2D. Furthermore, to our knowledge, this is the first study that examines specific proteins involved in fat metabolism and fuel utilization to shed light on the possible mechanisms by which exercise can prevent the HFD/OVX‐induced T2D.

## Materials and Methods

### Experimental groups

**Figure 1:**
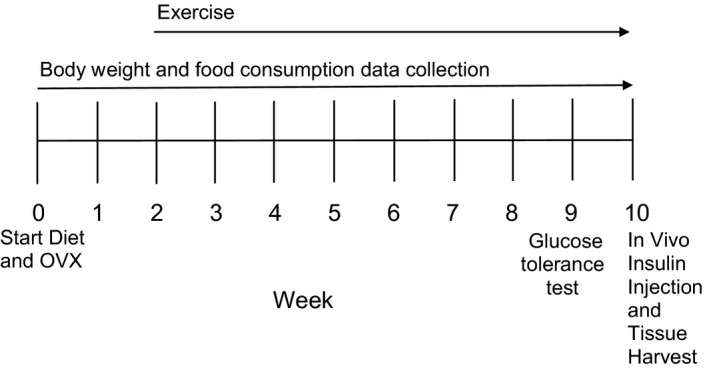
Timeline of the experimental design. At the start of the study (week 0), female Wistar rats were fed a standard diet (10% kcal fat) or a HFD (60% kcal fat). Some of the rats fed the HFD also underwent OVX. At week 2, a subset of the HFD/OVX rats experienced exercise training on a motor‐driven treadmill 5X per week. Body weight and food consumption was measured weekly. After 9 weeks, a glucose tolerance test was performed following an overnight fast. At the end of week 10, the fasted rats were given an insulin injection (i.p.), and the skeletal muscle (soleus and EDL) and periuterine WAT were harvested.

Female Wistar rats (Envigo) weighing 164 ± 2 g (8‐week‐old) were kept in a temperature controlled environment at 22 ± 2°C on a 12 h:12 h light:dark cycle. The rats were fed either a standard diet (SD, 10% kcal fat; Research Diets D12450J) or a high‐fat diet (HFD, 60% kcal fat; Research Diets D12492) for 10 weeks. Also at the start of the diet, some rats fed the HFD underwent bilateral ovariectomy (OVX) to remove both ovaries, or their ovaries remained intact (In). Furthermore, a subset of the HFD/OVX rats experienced treadmill exercise (Ex). Therefore, there were four experimental groups: SD/In, HFD/In, HFD/OVX, and HFD/OVX/Ex (*n* = 8/group). Body weight and food consumption was measured on a weekly basis. A summary of the research design is shown in Figure 1. All of the procedures were approved by the Institutional Animal Care and Use Committees at Augustana University and the Sioux Falls Veterans Affairs Health Care System.

### Exercise protocol

A subset of the HFD/OVX animals exercised on a motor‐driven treadmill 5 times per week. For the first 2 weeks after the OVX (weeks 0–1), the animals did not exercise to allow for recovery following the OVX surgery. The following 2 weeks (weeks 2–3), the animals were introduced to the treadmill for 15 min per day at 35 cm/s at a 5° incline (week 2) and 20 min per day at 45 cm/sec at a 5° incline (week 3). Finally, from weeks 4–10, the animals exercised for 25 min per day at 45 cm/sec at a 5° incline for the remainder of the study.

### Intraperitoneal glucose tolerance test and insulin measurements

Nine weeks after starting the diet, the rats were subjected to an intraperitoneal glucose tolerance test (GTT). After a 12 h fast, the rats were injected with glucose (2 g/kg; i.p.), and blood glucose levels were measured via a drop of tail blood on a glucometer (Accu‐Check Active) at 15, 30, 60, 90, and 120 min after the glucose injection. At each time point, tail blood was also collected in heparinized microcapillary hematocrit tubes. The tubes were sealed with critoseal and centrifuged for 5 min in a hematocrit tube centrifuge at room temperature. The plasma was collected and stored at −80°C until insulin levels were measured via an ELISA (ALPCO 80‐INSRTU).

### In vivo insulin injection and tissue harvest

Ten weeks after the start of the HFD, the rats were fasted overnight for 12 h. Next, the animals were injected with insulin (Humalog insulin lispro; 5 U/kg; i.p.). Ten minutes after the insulin injection, the rats were anesthetized with propofol (120 mg/kg; i.p.). Twenty minutes following the propofol injection, the soleus and extensor digitorum longus (EDL) muscles, periuterine WAT, and uteri were removed and weighed. The tissue was frozen in liquid nitrogen and stored at −80°C for future analysis.

### Western blot analysis

Approximately 75 mg (soleus and EDL) or 500 mg (WAT) of tissue was homogenized in cell extraction buffer (ThermoFisher FNN0011) supplemented with 200 mmol/L PMSF (Fisher BP231), 200 mmol/L NaF (Sigma S6776), 200 mmol/L sodium orthovanadate (Sigma S6508), and protease inhibitor cocktail according to the manufacturer's instructions (Sigma P‐2714) at a ratio of 75 mg soleus/EDL:750 *μ*L buffer and 500 mg WAT:750 *μ*L buffer. The homogenized samples were rotated at 4°C for 30 min and then centrifuged at 3000 rpm for 20 min at 4°C. The supernatant was removed, and the protein concentration was determined by the Bradford Bio‐Rad Protein Assay Kit II (Bio‐Rad 5000002). The samples were mixed with 4X Bolt LDS sample buffer (ThermoFisher B0007) and 10X Bolt sample reducing agent (ThermoFisher B0009) and heated to 70°C for 10 min according to the manufacturer's instructions. The samples (100 *μ*g protein) were ran on 8% or 12% Bolt bis‐tris gels (ThermoFisher) according to the manufacturer's instructions. The protein was transferred to a PVDF membrane using the Pierce Power Blotter, blocked for 1 h at room temperature in 5% milk in TBST, and then incubated in a primary antibody with gentle shaking on a rocker at 4°C overnight. The primary antibodies against acetyl‐CoA carboxylase (ACC; 3662), pAkt S473 (9271), Akt (9272), phospho‐(ser‐thr) Akt substrate (PAS‐160; 9611), pAS160 Thr642 (pTBC1D4; 8881), AS160 (TBC1D4; 2670), adipose triglyceride lipase (ATGL; 2138), fatty acid synthase (FAS; 3189), citrate synthase (14309), cytochrome c oxidase (COX) IV (4844), and *α*‐tubulin (9099) were purchased from Cell Signaling Technology and used according to the manufacturer's instructions. The primary antibodies against the glucose transporter 4 (GLUT4; 53566‐HRP), lipoprotein lipase (LPL; 373759) and perilipin (390169) and the secondary antibody mouse IgG kappa binding protein conjugated to horseradish peroxidase (m‐IgG*κ* BP‐HRP; 516102) were purchased from Santa Cruz Biotechnology and used according to the manufacturer's instructions. The secondary antibody peroxidase AffiniPure donkey anti‐rabbit IgG (H+L) (711‐035‐152) was purchased from Jackson Immuno Research and used according to the manufacturer's instructions. The proteins were detected using SuperSignal west pico PLUS chemiluminescent substrate (ThermoFisher 34577) and visualized using a UVP ChemStudio imager. The membrane was stripped with restore plus western blot stripping buffer (ThermoFisher 46430) for 15 min at 37°C according to the manufacturer's instructions. The membranes that were used to detect phosphorylated proteins were re‐probed for the total protein, and the membranes that were used to detect nonphosphorylated proteins were re‐probed for tubulin. The protein bands were quantified using ImageJ densitometry.

### Statistical analysis

The data were statistically analyzed using IBM SPSS Statistics version 24. Normality was determined using the Shapiro–Wilk test. Normally distributed data were analyzed using a one‐way ANOVA and a Tukey post‐hoc test. Non‐normally distributed data were analyzed using Kruskal–Wallis testing. The data are presented as the mean ± SE with *n* = 8/group.

## Results

### Exercise ameliorates the HFD/OVX‐induced Increase in body weight and WAT

**Figure 2:**
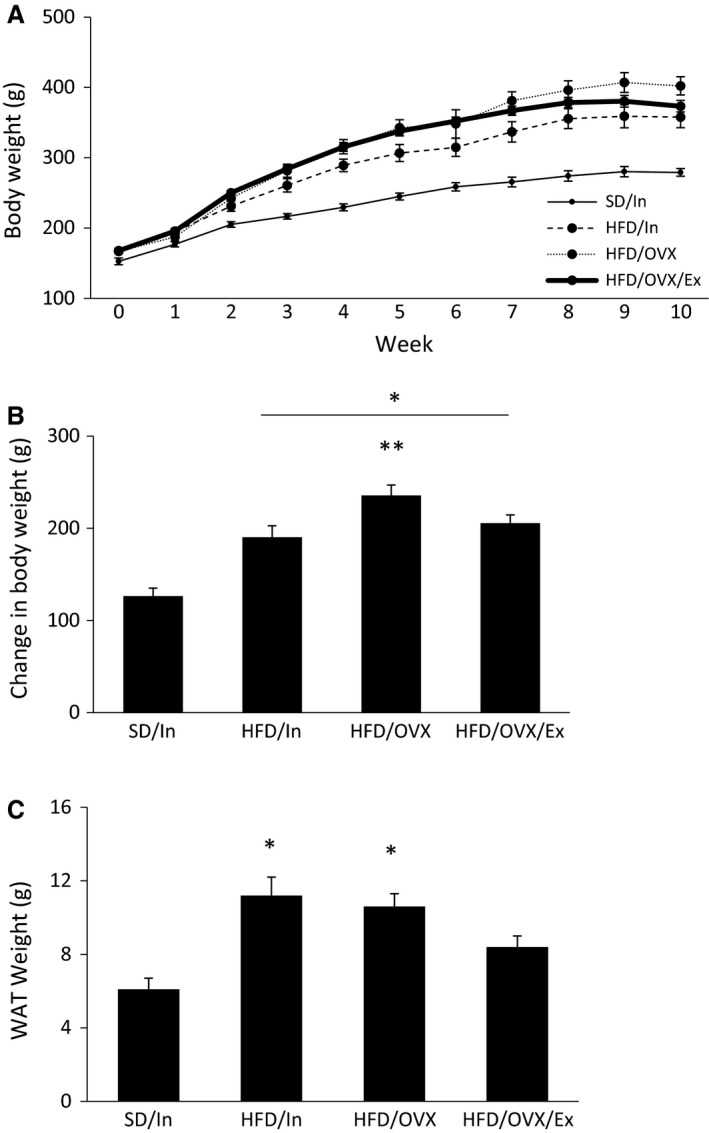
Exercise ameliorates total body weight gain and WAT weight gain. Body weight was measured on a weekly basis (A). The change in body weight was calculated as the difference in body weight between week 10 and week 0 (B). After week 10, the periuterine WAT was harvested and weighed (C). The data are presented as the mean ± SE. *P < 0.01 versus SD/In; **P < 0.05 versus HFD/In.

Throughout the 10 week study, the weekly body weights were noticeably greater in all groups consuming the HFD (Fig. 2A), and statistical analyses show that the change in body weight was significantly greater in all three groups fed the HFD compared to the SD group (*P *<* *0.01; Fig. 2B). Furthermore, the HFD/OVX group gained significantly more body weight compared to the HFD/In group (*P* < 0.05; Fig. 2B), suggesting that the presence of estrogens in the HFD/In group provides some protection against HFD‐induced weight gain. However, the HFD/OVX/Ex group did not weigh significantly more than the HFD/In group, even though estrogens were absent in the HFD/OVX/Ex group. These results suggest that exercise can effectively minimize the weight gain occurring with estrogen loss in the context of a HFD.

**Figure 3:**
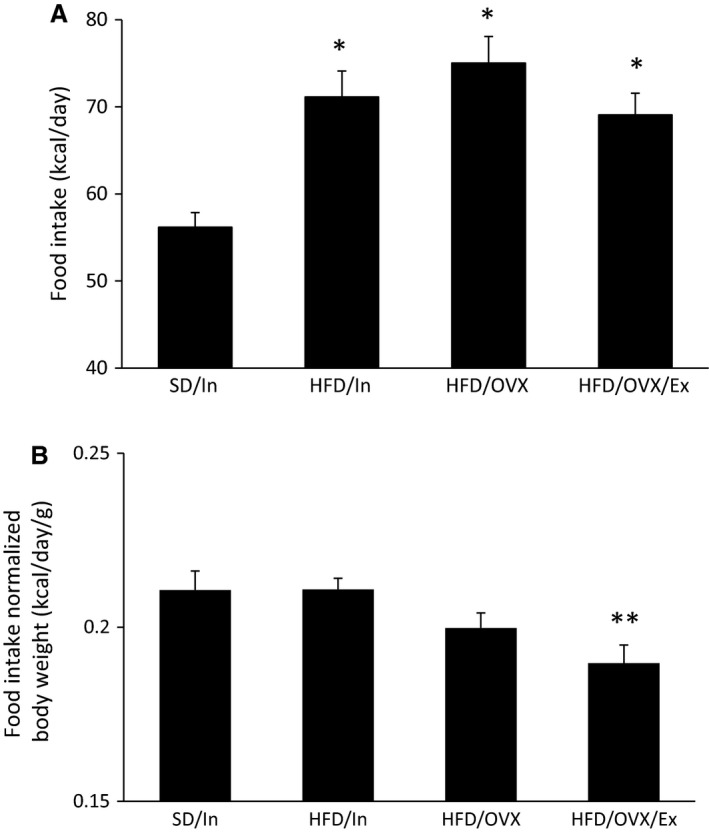
Consumption of a HFD increases the absolute kcal consumed per day (A). When normalized to body weight, the HFD did not increase the kcal consumed per day, and exercise significantly decreased the kcal consumed (B). The data are presented as the mean ± SE. *P < 0.01 versus SD/In; **P < 0.05 versus SD/In and HFD/In.

In addition to the changes in the overall body weight, the amount of adipose tissue in the abdominal area remains an important factor for T2D. The HFD/In and HFD/OVX groups gained significantly more WAT compared to the SD/In group (*P* < 0.01; Fig. 2C) with no difference between the HFD/In and HFD/OVX groups, suggesting that the absence of ovarian estrogens does not further increase the amount of periuterine WAT, although other fat depots were not measured. Notably, the HFD/OVX/Ex group did not have significantly more WAT compared to the SD/In group, suggesting that exercise is an effective way to prevent WAT weight gain, even when combining a HFD with OVX. The decreased body weight and WAT weight in the HFD/OVX/Ex group occurred even though the absolute caloric intake did not differ among the three HFD groups during the exercise treatment (weeks 4–10; Fig. 3A). Nonetheless, all three HFD groups consumed significantly more kcal per day compared to the SD/In group (*P* < 0.01; Fig. 3A). However, when normalizing the caloric intake to body weight, the food intake data show different results. The HFD/OVX/Ex group consumed significantly fewer kcal per day compared to the SD/In and HFD/In groups (Fig. 3B, *P* < 0.05), suggesting that exercise may suppress caloric intake.

The WAT contains aromatase and has the potential to produce estrogens. Thus, an increase in circulating estrogens may occur in tandem with increased WAT weight, even with OVX. The uterine weight is a reliable bioassay of circulating estrogens (Kendrick et al. 1987; Gorres et al. 2011; Benmansour et al. 2016). In the current study, the uterine weight of the SD/In, HFD/In, HFD/OVX, and HFD/OVX/Ex groups assessed at the time of euthanasia was 445.7 ± 36.8, 465.7 ± 32.3, 123.4 ± 11.3, and 118.0 ± 12.7 mg, respectfully. The OVX rats had a significantly lower uterine weight compared to the intact rats (*P* < 0.001), with no effect of diet, which is consistent with previous studies (Bryzgalova et al. 2008; Akamine et al. 2010). This confirms that the potential for estrogen production via the increased WAT weight was negligible in this study.

### Improvements in blood glucose with exercise

**Figure 4:**
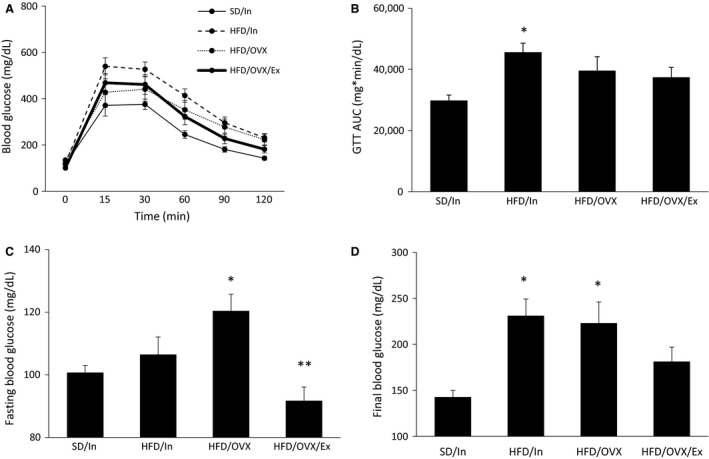
Exercise decreases hyperglycemia. After 9 weeks, the rats were fasted for 12 h prior to an intraperitoneal glucose tolerance test (GTT; (A)), and the area under the curve (AUC) was calculated (B). The fasting blood glucose levels (C) represent the blood glucose at time point 0 min of the GTT, and the final blood glucose levels (D) represent the blood glucose at time point 120 min of the GTT. The data are presented as the mean ± SE. *P < 0.05 versus SD/In; **P < 0.01 versus HFD/OVX.

To assess blood glucose regulation, we performed a GTT (Fig. 4A). The area under the curve (AUC) shows the overall ability of the animals to regulate blood glucose levels throughout the entire GTT. Although the HFD/In group had a significantly greater AUC compared to the SD/In group (*P* < 0.05; Fig. 4B), the HFD/OVX and HFD/OVX/Ex groups did not. The fasting blood glucose levels (time point 0 of the GTT) and the final blood glucose levels during the GTT are also clinical indicators of T2D. The HFD/OVX group had a significantly greater fasting blood glucose level compared the SD/In group (*P* < 0.05; Fig. 4C) but the HFD/In group did not, suggesting that the presence of estrogens provides a physiological benefit for fasting blood glucose levels. Additionally, the fasting blood glucose level of the HFD/OVX/Ex group was significantly less than the HFD/OVX group (*P* < 0.01; Fig. 4C). The results of the final blood glucose values were similar to that of the fasting levels in that the HFD/OVX group had a significantly greater value compared to the SD/In group (*P* < 0.05; Fig. 4D), whereas the HFD/OVX/Ex group did not. Additionally, the HFD/In group also had final blood glucose levels greater than that of the SD/In group (*P* < 0.05). Taken together, these data suggest that exercise can prevent the combined HFD‐ and OVX‐induced hyperglycemia.

### Exercise ameliorates HFD/OVX‐induced hyperinsulinemia

**Figure 5:**
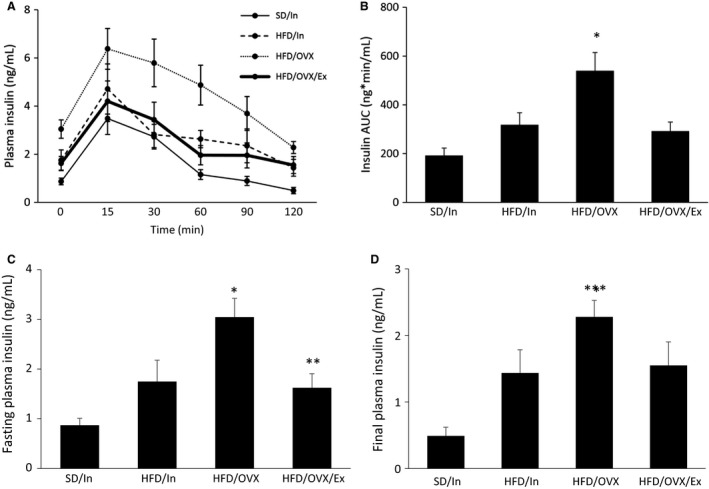
Exercise ameliorates the HFD/OVX‐induced hyperinsulinemia. During the GTT, tail blood was collected at each time point to measure the plasma insulin levels via an ELISA (A), and the area under the curve (AUC) was calculated (B). The fasting plasma insulin levels (C) represent the insulin levels at time point 0 min of the GTT, and the final plasma insulin levels (D) represent the insulin levels at time point 120 min of the GTT. The data are presented as the mean ± SE. *P < 0.05 versus all other groups; **P < 0.05 versus HFD/OVX; ***P < 0.05 versus SD/In.

Hyperinsulinemia is a classical indicator of T2D. Thus, the plasma insulin levels were measured during the GTT. The HFD/OVX group had noticeably greater insulin levels throughout the GTT, signifying that more insulin was needed to stimulate glucose removal from the blood (Fig. 5A). The statistical analysis shows that the AUC for the HFD/OVX group was significantly greater than all of the other groups (*P* < 0.05; Fig. 5B), and exercise was able to prevent this hyperinsulinemia. Additionally, the fasting insulin level was greater in the HFD/OVX group compared to all of the other groups (*P* < 0.05; Fig. 5C). However, the fasting insulin level of the HFD/OVX/Ex group was not significantly greater than the other groups and was even significantly lower than the HFD/OVX group (*P* < 0.05). Furthermore, at the end of the GTT, the plasma insulin level of the HFD/OVX group, but not in the HFD/In and HFD/OVX/Ex groups, remained significantly greater than the SD/In group (*P* < 0.05; Fig. 5D). These data suggest that when consuming a HFD, the presence of estrogens prevents hyperinsulinemia, but estrogen loss does result in hyperinsulinemia, which can be ameliorated with exercise.

### Insulin signaling is preserved even when combing a HFD with OVX

**Figure 6:**
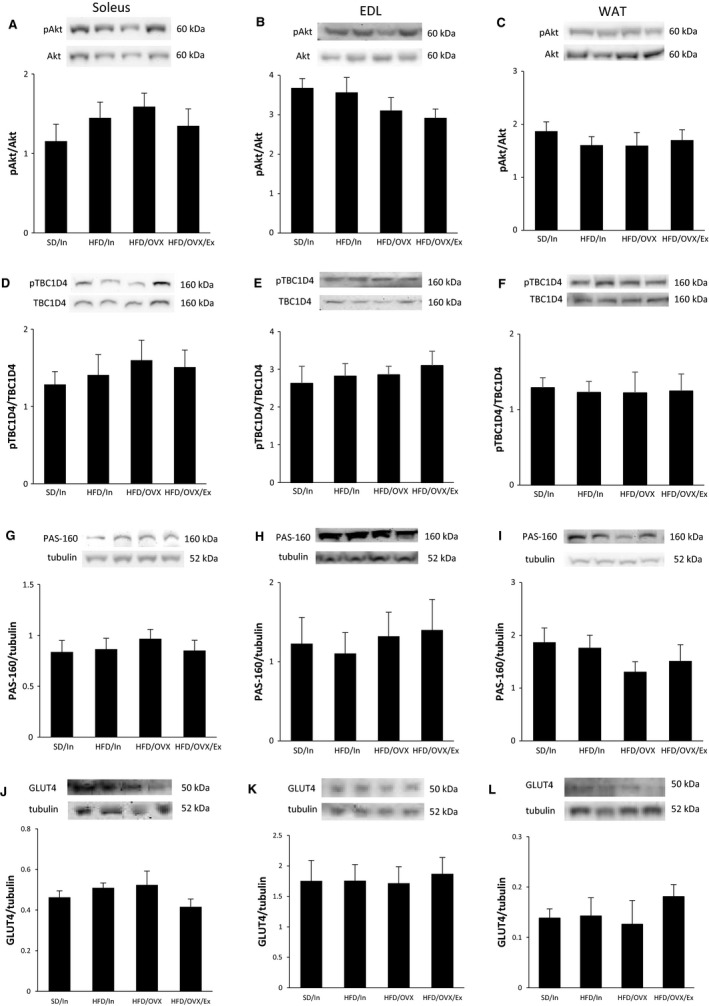
Insulin signaling is preserved even when combing a HFD with OVX. After 10 weeks, the rats were fasted for 12 h prior to an intraperitoneal insulin injection. The soleus, EDL, and periuterine WAT were harvested and frozen in liquid nitrogen. Western blot analysis measured activation of the insulin signaling pathway via pAkt (A–C), pTBC1D4 (D–E), and PAS‐160 (G–I) and total GLUT4 (J–L). The data are presented as the mean ± SE.

Numerous studies in male rodents fed a HFD demonstrate decreased activation of the insulin signaling pathway (Pedersen et al. 1991; Han et al. 1995; Zierath et al. 1997; Tremblay et al. 2001). Therefore, activation of the insulin signaling pathway was assessed in this study in the soleus (type I skeletal muscle), EDL (type II skeletal muscle), and abdominal WAT via Western blot analysis of pAkt, pTBC1D4, and PAS‐160 (Fig. 6A–I). Remarkably, the HFD did not decrease activation of the insulin signaling pathway in the skeletal muscle or WAT, even when combined with OVX. These results are in stark contrast with the well‐established knowledge in the literature that a HFD induces defects in the insulin signaling pathway. However, much of this well‐established knowledge was concluded from studies using male rodents.

At the end of the insulin signaling pathway, the glucose transporter 4 (GLUT4) translocates and inserts into the plasma membrane of skeletal muscle and WAT to allow glucose uptake into the tissue. Even when insulin signaling is preserved, a decrease in total GLUT4 may result in decreased glucose uptake into the tissue. Therefore, the total GLUT4 protein content was measured in the current study. However, no changes in total GLUT4 occurred in the soleus, EDL, or WAT (Fig. 6J–L).

### Alterations in fat metabolism

**Figure 7:**
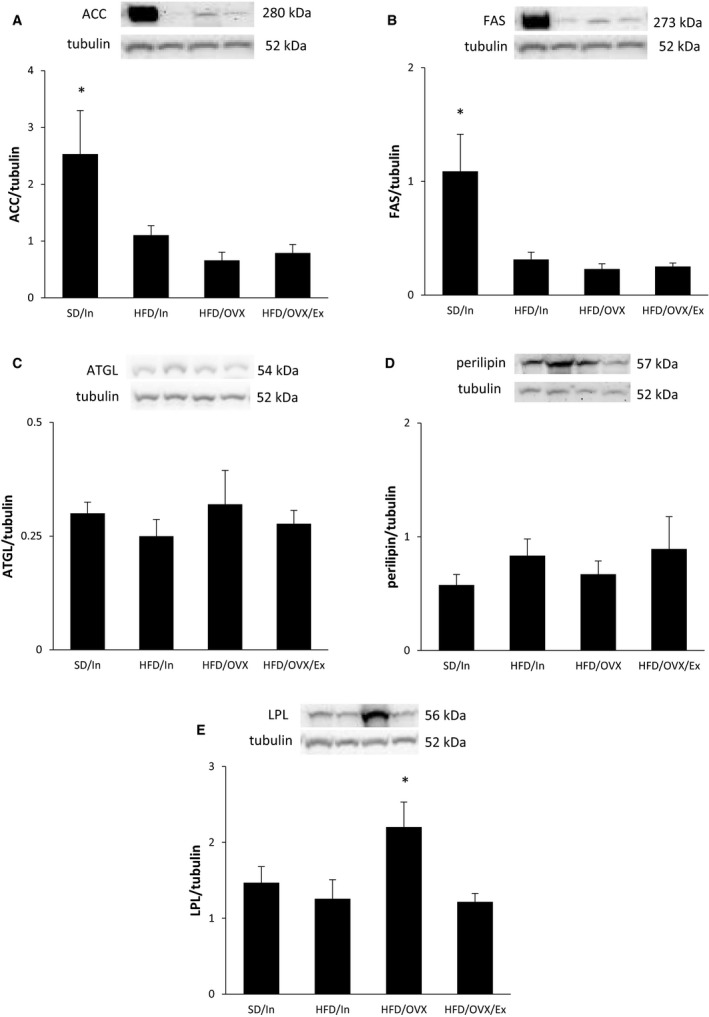
Alterations in proteins involved in fat metabolism. Western blot analysis of the periuterine WAT measured the level of proteins involved in de novo lipid synthesis (A–B), lipolysis (C–D), and fat storage (E). The data are presented as the mean ± SE. *P < 0.05 versus all other groups.

Proteins involved in fat metabolism were assessed in the WAT to explain the total body and WAT weight changes. ACC and FAS are two protein involved in de novo lipid synthesis. These proteins were significantly greater in the SD/In group compared to all three groups fed the HFD (*P* < 0.05; Fig. 7A–B). Thus, the consumption of a HFD appears to downregulate de novo lipid synthesis, regardless of ovarian status or exercise. While there were no significant differences in the lypolytic proteins ATGL and perilipin (Fig. 7C–D), the increased WAT weight in the HFD/OVX group may be due to increased LPL expression, a protein that simulates fat storage (*P* < 0.05; Fig. 7E). Notably, exercise prevented an increase in LPL expression, which may account for the decreased WAT weight in the HFD/OVX/Ex group.

### HFD/OVX decreases the function of the krebs cycle, but not oxidative phosphorylation

**Figure 8:**
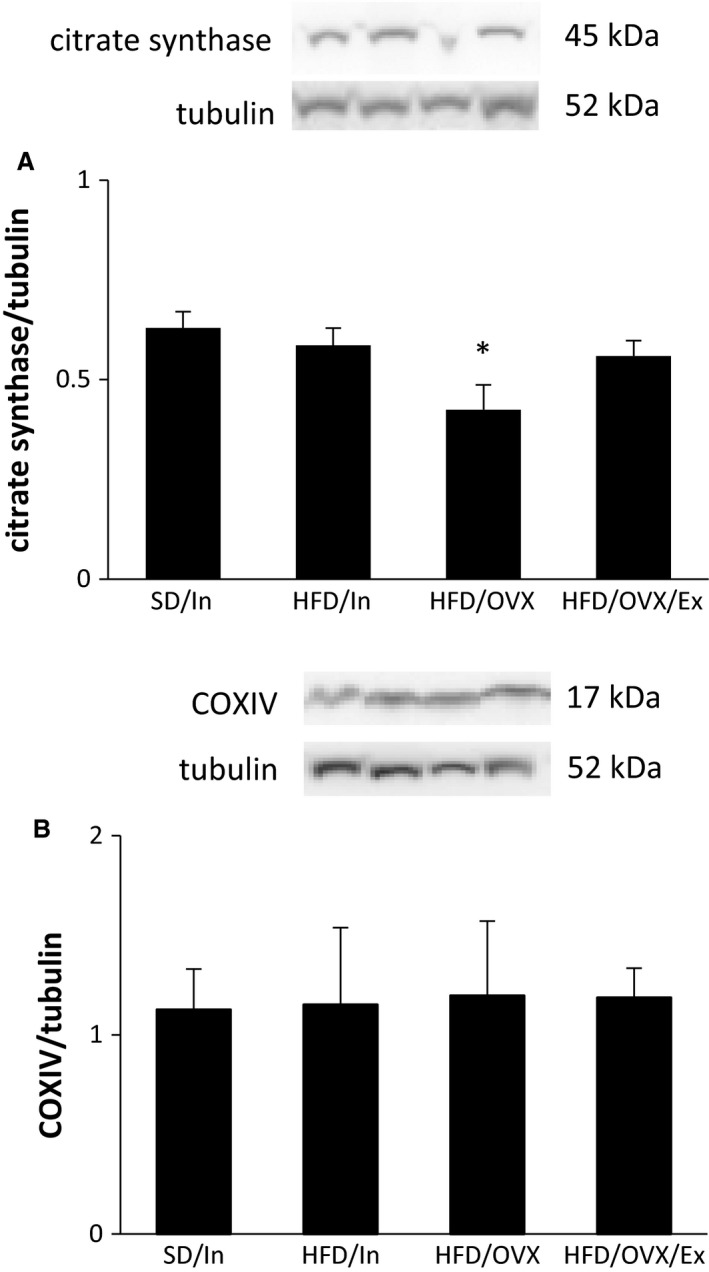
The HFD/OVX decreases the function of the Krebs cycle, but not oxidative phosphorylation. Western blot analysis of the periuterine WAT measured the levels of proteins involved in the Krebs cycle (A) and oxidative phosphorylation (B). The data are presented as the mean ± SE. *P < 0.05 versus SD/In.

Mitochondrial proteins in the WAT were also measured to assess the Krebs cycle and oxidative phosphorylation. Citrate synthase, an enzyme involved in the Krebs cycle, did not change in the HFD/In rats but was significantly decreased in the HFD/OVX group (*P* < 0.05; Fig. 8A). Moreover, the citrate synthase level in the HFD/OVX/Ex group was restored. These data suggest that the presence of ovarian estrogens may provide benefits by improving fuel utilization, and exercise offers valuable benefits in the absence of ovarian estrogens. No changes occurred in COXIV, a protein involved in oxidative phosphorylation (Fig. 8B). Furthermore, no changes occurred in the citrate synthase and COXIV protein level in the soleus and EDL (data not shown). Certainly, more studies are needed to definitively determine the functional changes in the Krebs cycle and oxidative phosphorylation in the WAT in female rodents fed a HFD with and without OVX and exercise, particularly studies directly measuring mitochondrial enzymatic activity in the WAT.

## Discussion

Overall, this study provides novel information regarding exercise's ability to prevent T2D risk factors and on the regulation of proteins involved in fat metabolism and fuel utilization in the context of a HFD combined with OVX. While previous studies show exercise's ability to prevent T2D in OVX rodents (Saengsirisuwan et al. 2009; MacDonald et al. 2015), this study adds valuable information for the omnipresent physiological state that combines a HFD with the loss of ovarian estrogens, which together provide significant physiological challenges. A previous study demonstrates the ability of exercise to decrease the total body weight and visceral fat weight in the context of a HFD with OVX (Zoth et al. 2012), but the specific metabolic pathways (such as insulin signaling, fat metabolism, and fuel utilization) were not assessed. One limitation of the current study and other similar studies mentioned throughout the discussion pertains to the potential for treadmill running to induce stress. The treadmill running‐induced stress was not controlled for, and thus, the effects of exercise may be due to stress hormones.

The beneficial effects of estrogens on insulin action and glucose homeostasis are supported by studies showing greater insulin sensitivity in premenopausal women compared with age‐matched men (Nuutila et al. 1995; Donahue et al. 1997; Nilsson et al. 2000; Borissova et al. 2005; Vistisen et al. 2008; Hoeg et al. 2009; Karakelides et al. 2010), lower fasting glucose in females (Nilsson et al. 2000; Karakelides et al. 2010), and lower insulin secretion in females during a GTT (Donahue et al. 1997). However, these benefits are lost after menopause, as postmenopausal women have higher fasting blood glucose and insulin compared to age‐ and body mass index‐matched premenopausal women (Lynch et al. 2002) and decreased insulin sensitivity and glucose tolerance (Lindheim et al. 1994). To account for the differences between pre‐ and postmenopausal women, assessing the change in body weight, and in particular the abdominal adipose tissue weight, is valuable information because abdominal obesity is the number one risk factor for T2D (Kissebah and Peiris 1989; Chan et al. 1994; Despres 2006).

Consistent with our data, Weigt et al. (2012) show that combining a HFD with OVX increases the overall body weight gained compared to a HFD alone; however, exercise was not assessed as a treatment. Therefore, our study adds to the literature regarding exercise's ability to prevent weight gain during the combined HFD/OVX physiological state. However, in contrast with our data showing increased periuterine WAT in the HFD/In rats, Weigt et al. (2012) did not see an increase in the visceral adipose tissue weight in intact rats fed a HFD compared to intact rats fed a standard diet; however, the visceral adipose tissue weighed in their study included the periovarian, perirenal, and mesenteric/omental fat pads, while our study assessed the periuterine fat. Additionally, when considering that our data show a significant increase in total body weight in the HFD/OVX group compared to the HFD/In group, but no difference between the two groups’ periuterine WAT weight, considering the change in the overall percent body fat and/or the specific changes in each fat pad deserves further attention to clarify the role of estrogens in specific fat pad regulation. When considering exercise as a treatment, the length of exercise treatment appears important as a previous study shows that 6 weeks of exercise training decreased the visceral adipose tissue weight in OVX rats fed a HFD, but not the overall body weight (Zoth et al. 2010). In our study, 8 weeks of exercise training decreased both the total body weight and the WAT weight.

When pinpointing the factors contributing to the body weight and WAT weight changes, caloric intake and caloric expenditure are two key components to consider. A previous study shows that OVX increased the caloric intake in rats fed a standard diet and a HFD (Gorres et al. 2011). However, other studies, including the present study, did not find increased caloric intake with ovarian hormone loss (Rogers et al. 2009; Duval et al. 2014; Vieira‐Potter et al. 2015).

One major limiting factor of the current study is that the total energy expenditure was not measured. While the current study prescribed exercise to HFD/OVX rats, the energy expenditure due to voluntary cage activity was not assessed among the groups. Notably, studies demonstrate that ovarian hormone loss can reduce voluntary activity (Rogers et al. 2009; Izumo et al. 2012; Duval et al. 2013; Vieira‐Potter et al. 2015). Therefore, in the present study, the increased weight gain with OVX may be due to decreased voluntary cage activity, despite equal caloric intake between the HFD/In and HFD/OVX rats.

Presuming that estrogens provide protective effects against HFD‐induced T2D, we hypothesized that the GTT AUC for the HFD/OVX group would be greater than the HFD/In group. However, this effect was not shown in the present study and in a previous study (Gorres et al. 2011). We suggest that the hyperinsulinemia in the HFD/OVX may be responsible for preventing robust hyperglycemia, and the following studies support this theory. While MacDonald et al. (2015) demonstrated that OVX alone can increase the GTT AUC after 10 weeks, Saengsirisuwan et al. (2009) did not see an increase in the GTT AUC 12 weeks post‐OVX. The increased GTT AUC in the MacDonald et al. study can be explained by the lack of hyperinsulinemia; whereas the rats in the Saengsirisuwan et al. study did show hyperinsulinemia (via increased insulin AUC), which likely contributed to the lower glucose levels during the GTT. Therefore, when assessing and interpreting changes in blood glucose levels, the insulin levels should be considered as well.

Exercise can prevent the OVX‐induced hyperglycemia in the context of a standard diet (MacDonald et al. 2015), and 6 weeks of exercise training in OVX rats fed a HFD tended to decrease serum insulin levels (although this trend was non‐significant) (Zoth et al. 2010). For the first time, our study shows that the combination of a HFD/OVX for 10 weeks can induce hyperglycemia (during fasting and at the end of the GTT) and hyperinsulinemia (during fasting and throughout the GTT), and exercise treatment for 8 weeks can ameliorate both of these deleterious effects.

Notably, we did not see any defects in the insulin signaling pathway in type I (slow‐twitch) skeletal muscle (soleus), type II (fast‐twitch) skeletal muscle (EDL), or WAT. These results are markedly different from several studies in male rats fed a HFD showing a decrease in the insulin signaling pathway and glucose uptake in the skeletal muscle and adipose tissue (Pedersen et al. 1991; Han et al. 1995; Zierath et al. 1997; Tremblay et al. 2001), but are in agreement with previous studies in female rats showing no defects in the insulin signaling pathway and glucose uptake in the skeletal muscle and adipose tissue after high‐fat feeding combined with OVX (Gorres et al. 2011) or OVX alone (MacDonald et al. 2015). In human studies, the increased whole body insulin sensitivity in women can be explained by greater glucose uptake into the skeletal muscle (Nuutila et al. 1995; Hoeg et al. 2009). Thus, the insulin signaling pathway in female skeletal muscle may be better suited to respond to insulin and may also possess resilience against defects in the signaling pathway, although more studies are needed to confirm this hypothesis. Alternatively, while the in vivo insulin injection protocol used in the current study is widely used throughout the field (Ritchie et al. 2014; Hamilton et al. 2015; MacDonald et al. 2015), many signaling pathways, including the insulin signaling pathway, are time‐dependent. Therefore, the absences of changes in the current study may be due to assessing a single time point following the insulin injection.

For the first time, the present study adds novel information regarding proteins involved in fat metabolism and fuel utilization. In a previous study, forty days of estradiol treatment in OVX mice fed a standard diet decreased LPL and ACC expression in the adipose tissue compared to OVX mice without estradiol treatment (D'Eon et al. 2005). In our study, we did not see any differences in WAT LPL and ACC expression between intact and OVX rats fed a HFD. Therefore, the HFD may override the ability of estrogens to regulate de novo fat synthesis, which is consistent with previous data (Weigt et al. 2013). We furthermore show that either exercise does not affect the LPL and ACC protein levels or that the consumption of the HFD diet also overshadows any potential effects of exercise. A previous study found that the lipolytic genes perilipin and hormone sensitive lipase (which has a similar function as the ATGL measured in the current study) did not change in OVX mice with and without estradiol replacement who were fed a standard diet (D'Eon et al. 2005). Our study supports this data and also adds that lipolysis does not change even when consuming a HFD and in the context of exercise. Our data is also consistent with a previous report showing that combining a HFD with OVX increases the expression of the fat‐storing protein LPL in the WAT (Weigt et al. 2013), and our study shows for the first time the ability of exercise to decrease LPL in OVX rats fed a HFD, which may contribute to the decreased WAT weight in the HFD/OVX/Ex group in this study.

The ability of the mitochondria to utilize fuel remains an important factor in body weight and WAT weight regulation. Therefore, we assessed citrate synthase, the rate limiting enzyme in the Krebs cycle, and COXIV, an enzyme in the electron transport chain, in the WAT. Our study shows no changes in COXIV protein levels, and this result is mirrored by a study showing no changes in COXIV in the visceral adipose tissue with OVX alone, although OVX alone resulted in decreased COXIV in the subcutaneous adipose tissue (MacDonald et al. 2015). While a previous report shows that OVX alone does not decrease citrate synthase in the visceral adipose tissue (MacDonald et al. 2015), we show that when combining OVX with a HFD, citrate synthase does decrease, and exercise is able to restore this level. Furthermore, the HFD/In rats did not show decreased citrate synthase; thus, when consuming a HFD, the presence of ovarian estrogens may provide protection by promoting fuel utilization in the mitochondria. Taken together, this information provides novel insights into the physiological state that combines a HFD and OVX and the ability of exercise to prevent the deleterious effects in fuel utilization.

Although previous studies show the ability of exercise to combat OVX‐induced T2D, this study, for the first time, assesses the ability of exercise to combat T2D in the combined, deleterious physiological state of a HFD and OVX and assesses the insulin signaling pathway and proteins involved in fat metabolism and fuel utilization. This study clarifies the impact of exercise on the HFD/OVX‐induced increase in body weight and WAT weight, suggesting that exercise is effective at decreasing both, but future studies should consider the changes in several fat depots to clarify the role of estrogens in specific fat pad regulation. This study also elucidates the discrepancies in the literature regarding whether or not changes in blood glucose levels occur. Particularly, the insulin levels throughout the GTT are of utmost importance to measure and consider when assessing and interpreting blood glucose levels. Finally, this study provides novel information regarding proteins involved in fat metabolism and mitochondrial fuel utilization. For the first time, we show that combining a HFD and OVX results in an increase in the fat‐storing protein LPL and a decrease in the Krebs cycle enzyme citrate synthase. Notably, exercise combats both of these changes, suggesting that exercise acts as a prophylactic treatment for T2D by decreasing the fat storage capability and increasing the Krebs cycle's ability to use energy, even when combining two challenging physiological states.

## Conflict of Interest

No conflicts of interest, financial or otherwise, are declared by the author(s).
